# 1,4-Dihydropyridines: The Multiple Personalities of a Blockbuster Drug Family

**Published:** 2012-10-11

**Authors:** Mauro Cataldi, Fiorentina Bruno

**Affiliations:** Division of Pharmacology, Department of Neuroscience, Federico II University of Naples, Naples, ITALY

**Keywords:** dihydropyridines, voltage-gated Ca^2+^ channels, enantiomers, L-type channels, T-type channels

## Abstract

More than 40 years after their introduction in therapy, 1,4-dihydropyridines (DHPs) are still amongst the most prescribed drugs in the world. Though they all share a similar mechanism of action blocking L-type voltage-gated Ca^2+^ channels, DHPs differ in crucial pharmacological properties like tissue selectivity and cardiodepressant activity. This review examines how changes in the DHP structure can modify the pharmacological properties of these drugs and how some of these chemical manipulations have been exploited to obtain clinically more effective molecules. Special emphasis is given to the evidence that L-type Ca^2+^ channels are an heterogeneous family and that DHPs with different pharmacological properties differ in their affinity for the different isoforms of this class of channels. Data showing that DHP pharmacological heterogeneity could be in part dependent on the interaction of some of these molecules with ion channels different from the L-type Ca^2+^ channels is reviewed as well.

## I. INTRODUCTION

Since their introduction in therapy more than 40 years ago [1], 1,4-dihydropyridines (DHPs) have been amongst the most successful drugs ever used in humans. Testifying this undeniable success amlodipine ranks amongst the 10 most prescribed drugs in the United States [2] as in the rest of the world. Like phenylalkilamines and benzothiazepines, DHPs act by blocking L-type voltage gated Ca^2+^[3]. These ion channels are multimeric protein composed by a pore forming α1 subunit and by accessory α2δ, β and, variably, γ subunits [4,5]. Among their many functional roles, L-type Ca^2+^ channels are crucial in controlling heart contractility and excitability [6], vascular tone [7] and the generation of spontaneous depolarizations in cardiac, neuronal or endocrine cells with pacemaking activity [8–10].

DHPs differ from the other Ca^2+^ channel blockers because of their marked selectivity for vascular smooth cells respect to myocardium. This selectivity confers to DHPs the property of being good antihypertensive drugs with small or no cardiodepressant activity [11]. Two different mechanisms have been proposed to explain the higher DHP activity on blood vessels as compared with the heart: 1- an higher state-dependent affinity for the inactivated forms of L-type channels in vascular smooth muscle cells, and 2- the existence of two different isoforms of these channels, a cardiac isoform, exuisitely sensitive to phenylalkilamine inhibition, and a vascular smooth muscle cell isoform, preferentially inhibited by DHPs. The latter hypothesis has been formally demonstrated when different splicing variants of the Ca_V_1.2 channel gene expressed in the heart and in vascular smooth muscle cells were identified [12–14]. The cardiac (Ca_V_1.2a) and smooth muscle (Ca_V_1.2b) isoforms differ in four different splicing loci: exon 1/1a in the N-terminus, exon 8/8a in the transmembrane segment IS6, exon 31/32 in the transmembrane segment IVS3, and exon 9* in the loop that connects domains I and II. Specifically, the exon composition of the cardiac and vascular smooth muscle cell isoform are the following: 1a/8a/Δ9*/31/33 * [15], and 1/8/9*/ 32/33 [16], respectively. Importantly, when expressed in heterologous systems these two isoforms showed the different sensitivity to DHPs and other Ca^2+^ channel blockers observed in the heart and in blood vessels; in addition, mutagenesis studies showed that exon 8 in the IS6 region of the channel dictates DHPs selectivity [13–14].

Concerning the first hypothesis, i.e. that DHP could more active on vascular ion channels because they preferentially block inactivated Ca^2+^ channels in vascular smooth muscle cells, experiments performed with the cloned cardiac and vascular isoforms showed that gating differences cannot explain DHP tissue selectivity [17]. However, more recently, a splice variant that differs from the canonical Ca_V_1.2b isoform because it lacks exon 33 (Ca_V_1.2SM: 1/8/9*/ 32/Δ33) was identified in vascular smooth muscle cells and it was shown to be specifically sensitive to state-dependent inhibition by nifedipine [18]. Therefore, the vascular districts expressing the Ca_V_1.2SM isoform, DHPs could specifically display a more marked state/dependent block of L-type Ca2+ channels.

A number of excellent reviews have been published on the structure, mechanism of action and clinical uses of DHPs [for instance, 19–20]. Here, instead, we will focus on an interesting characteristic of this drug family, that has not been so extensively addressed in the literature: its pharmacological heterogeneity. While, indeed, all DHPs share a common molecular backbone and act on similar molecular targets, important differences do exist among them both in their pharmacokinetic and pharmacodynamic properties. It has been proposed that the choice of the more appropriate DHP in specific clinical settings should take into account these differences that could also could influence the safety of each of these molecules.

A first relevant pharmacodynamic difference among DHPs pertains tissue selectivity as classical observations showed that some DHPs could be more effective in relaxing some vascular beds than others. In particular, manidipine is relatively specific for renal vessels and seems to be a good choice in patients in which the preservation of a deranged renal function is the primary concern [21–22] and the same has been reported also for benidipine, efonidipine and nivaldipine [23]. In addition, nimodipine has been proposed to be quite selective for brain vessels and is recommended for the treatment of the vasospasm caused by subarhacnoid hemorragy [24–29], flunarizine is much more active on mesenteric artery than on the aorta [30] while nisoldipine is thought to be a relatively specific coronary vasorelaxant drug [31]. A second relevant pharmacodynamic difference concerns the effect on heart rate. Because of their predominant effect on blood vessels and of the fall in blood pressure that they cause, DHPs induce reflex tachycardia. However, important differences have been reported amongst the different members of this drug family in the entity of this tachycardic response. Specifically, isradipine and amplodipine are considered as the less tachycardic DHPs [32–34] and more recently cilnidipine was added to this list [35].

Finally, some DHPs retain some negative inotropic activity that can be harmful especially when significant plasma concentrations are rapidly reached [36]. This eventuality appears more consistent with drugs of short half-life like nifedipine whose clinical use has found to be associated to an increase in the incidence of sudden deaths or cerebral ischemia [37–38]. DHPs differ, indeed, also in their pharmacokinetics and, in particular, in their half life. Taking into account both their half-life and cardiodepressant properties, DHPs have been traditionally classified in three different groups, or generations: cardiodepressant DHPs with a short half-life, cardiodepressant DHPs with a long half-life and non cardiodepressant DHPs with a long half life [39–40]. In Tab. 1 this classification has been modified to introduce a new group of non-cardiodepressant DHPs with ultra-short half-life that is exemplified by clevidipine. This new DHP, which has been approved by FDA in 2008, represents an important advancement in the treatment of hypertensive emergencies because it couples the lack of cardiodepressant activity with a very favourable pharmacokinetic profile with a rapid onset and disappearance of its effects [41–43].

Considering that all DHPs share a similar mechanism of action as they block L-type Ca^2+^ channels, the question arises of establishing how and why different DHPs could be pharmacologically different. While this question is still open and object of investigation by several groups, the (few) data available seem to suggest that specific substitutions at key residues of the DHP backbone could affect the specificity of these drugs for Ca_V_1.2 channels. Intriguingly, DHP pharmacological profile can be significantly affected by structural changes lowering drug specificity an conferring them some activity on ion channels different from Ca_V_1.2.

## II. DIFFERENT PHARMACOLOGICAL PROPERTIES OF DIFFERENT DHPs: JUST A MATTER OF ION CHANNEL SELECTIVITY?

### A- THE CASE OF NON-TACHYCARDIC DHPS

**Fig. 1:**
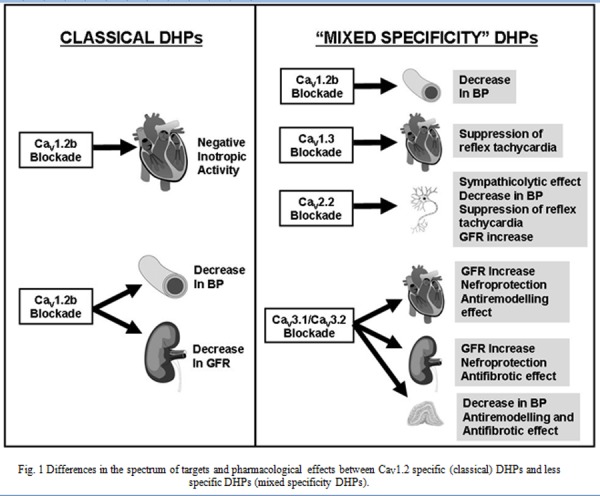
Differences in the spectrum of targets and pharmacological effects between Ca_V_1.2 specific (classical) DHPs and less specific DHPs (mixed specificity DHPs).

Isradipine, amlodipine and cilnidipine are a good example of how a relative loss of selectivity could be responsible for the better pharmacological profile of some DHPs as compared with the other members of this family. As mentioned above, these DHPs have been described as less tachycardic than classical DHPs. This important pharmacological property can be explained on the basis of their interaction with ion channels other than those encoded by the Ca_V_1.2 gene. In particular, two other voltage-gated Ca^2+^ channel subtypes seem to be involved, the Ca_V_1.3 and the Ca_V_2.2-encoded channels. The Ca_V_1.3 gene encodes for the pore forming subunit of the endocrine form of L-type Ca^2+^ channels, formerly known as α_1D_[44]. Ca_V_1.3 has a different distribution in the heart as compared with Ca_V_1.2. While, indeed, Ca_V_1.2 channels localize both in atria and in ventricles, Ca_V_1.3 channels are expressed only in atria [45] and represent the main L-type channel form in sinoatrial node [46]. Ca_V_1.3 channels differ from Ca_V_1.2 channels because of a more negative threshold for activation and of a less marked voltage-dependent inactivation [46–47]. Because of these biophysical properties Ca_V_1.3 channels are well suited to take part in the generation of repetitive depolarizations in pacemaker cells. Consistent with these data, severe bradyarrhythmias do occurr in Ca_V_1.3 knock-out mice suggesting that this channel isoform is critical for cardiac pacemaking and for maintaining sinusal rhythm [10, 46, 48–50]. Importantly, this hypothesis is also supported by the results of electrophysiological studies performed on isolated SAN and AVN cells [10, 46, 48–50]. While Ca_V_1.3 channels have been shown to be less sensitive to DHPs than Ca_V_1.2 channels [47], significant differences do exist among the different members of the DHP family as isradipine, amlodipine and azidopine are at least 3–4 times more effective than nifedipine or nitrendipine in blocking Ca_V_1.3 channels in vitro [51]. These results suggest that L-type Ca^2+^ currents in the sinoatrial and atrioventricular nodes are significantly blocked when either isradipine or, possibly, amlodipine are administered to hypertensive patients and this could explain why these drugs do not cause a significant reflex tachycardia (Fig. 1). Thus, specific DHPs may affect heart rate without exerting a significant negative inotropic activity. Confirming the hypothesis that negative inotropic and negative chronotropic effects could be dissociated in DHPs, among the different imidazo[2,1-b]thiazole DHP derivatives synthesized by Budriesi et al. [52] some have a marked negative inotropic activity others a marked negative chronotropic activity and others both. Interestingly, the ability to differentially affect contractility and rhythmicity in the myocardium has been demonstrated also in non-DHP compounds with L-type Ca^2+^channel blocking properties [53].

N-type channels Ca^2+^ channels whose pore forming unit is encoded by the Ca_V_2.2 gene are the other channel type whose blockade could explain why some DHPs are less tachycardic than others. These Ca^2+^ channel subclass, is expressed in neuronal axon terminals where it controls the release of neurotransmitter including glutamate [54], γ-aminobutyric acid [55], acetylcholine [56], dopamine [57], and noradrenaline [58]. They have a special relevance in cardiovascular physiology as they control noradrenaline release in the orthosympathetic nervous system. This is demonstrated by the ability of the N/type blockers ω-conotoxin GVIA and ω-conotoxin MVIIA (SNX-111) to inhibit noradrenaline release from superior cervical ganglia in vitro [59] and to exert a sympatholytic activity in vivo [60], respectively. A formal demonstration that N-type channel activity is essential for the orthosympathetic control of the cardiovascular system came from the evidence of its significant impairment in Ca_V_ 2.2 knock-out mice [61].

As shown by patch clamp experiments performed in heterologous expression systems with cloned Ca_V_2.2 channels the vast majority of DHPs are virtually ineffective on N-type channels. However, a small DHP subgroup consisting of amlodipine, benidipine, cilnidipine, nicardipine, and barnidipine seems to stand apart from the others for it exerts some blockade of this channel type [62]. N-type channel blocking activity seems to be relevant for amlodipine ad cilnidipine with IC_50_s close to those for L-type channel blockade and in the low micromolar range [62]. Importantly, evidence that the orthosympathetic system is actually blocked in vivo has been reported for amlodipine and cilnidipine as these DHPs both decrease serum catecholamine levels and decrease heart rate variability in humans [63–66]. The ability of some DHPs like cilnidipine or amlodipine to exert a sympathicolytic activity could be extremely helpful in improving the antihypertensive activity of these drugs as it not only prevents reflex tachycardia but also other unwanted consequences of reflex authonomic activation like secondary hypereninemia of reflex peripheral vasoconstriction (Fig. 1). While the real relevance of N-type Ca^2+^ channel blockade in determining the clinical activity of amlodipine remains doubtful, some evidence that it could be crucial in the case of cilnidipine has been provided [35]. Before proceeding further we have, however, to remind that drug pharmacokinetic could also greatly influence the tachycardic reflex response elicited by DHP. DHPs with a long half-life only slowly reach effective plasma concentrations and, therefore, do not cause the sudden changes in blood pressure as those needed to trigger reflex tachycardia. This may be important in the case of amplodipine, which has a very long half-life of about 30 hours [67].

### B- THE CASE OF “NEFROPROTECTIVE”DHPS

The small group of “nefroprotective” DHPs, including efonidipine, benidipine or manidipine, gives us an additional example of how some loss of selectivity could lead to DHPs with pharmacological properties different from those observed in more Ca_V_1.2-selective members of this family. Compelling experimental evidences suggest, indeed, that the ability of this group of DHPs to preferentially increase renal blood flow is the consequence of their ability to significantly block another subclass of voltage/gated Ca^2+^ channels, the T-type channels. Also known as low-voltage activated Ca^2+^ channels this channel family differs from HVA channels because of their permeation properties and because they open at much more negative potentials, inactivate more rapidly and deactivate more slowly [68–69]. Three different isoforms of T-type channels have been identified, Ca_V_3.1, Ca_V_3.2 and Ca_V_3.3 which differ in their electrophysiological properties and tissue d istribution [69]. T-type channels also differ from L-type channels in their pharmacology. Though really selective T-type blockers are still missing, these channels are blocked by insect toxins like kurtoxins and Pro/Tx-I [70–71], by neuroleptics [72], kinase inhibitors like imatinib mesylate [73] and non-steroidal anti-inflammatory drugs [74]. In addition, their gating behavior is profoundly affected by transition metals like Zn^2+^[75]. While the majority of the DHPs are ineffective or only minimally effective on the different T-type isoforms, some of them strongly block these channels at concentrations close to those effective on L-type channels. Furukawa et al. [76] performed a systematic analysis of the effects of several DHPs on Ca_V_3.1, Ca_V_3.2 and Ca_V_3.3 channels heterologously expressed in Xenopus oocytes. Among the many DHPs tested, the most potent in blocking T/type channels were barnidipine, manidipine and amlodipine that, at 10 μM concentrations, caused a 50% or higher current blockade; though less potent, also efonidipine, benidipine, and the old DHPs nifedipine and nicardipine displayed some T-type blocking effect [76]. Important differences were noted in the effect of these DHPs on the different Ca_V_3 isoforms. In general, the above mentioned DHPs were more effective on Ca_V_3.1 and Ca_V_3.2 channels whereas they induced only a slight Ca_V_3.3 blockade [76].

In addition, some DHPs like azelnipidine were more effective on Ca_V_3.1 whereas others like manidipine were significantly more effective on Ca_V_3.2 than on Ca_V_3.1 channels. Interestingly, nimodipine was almost ineffective on Ca_V_3.1 but significantly inhibited Ca_V_3.2 channels. The rank of T-type blocking activity by different DHPs reported by Furukawa, however, may be highly dependent on the expression system used because different results were obtained when DHP effect on these channels were evaluated in HEK cells [77]. In particular, in these cells efonidipine, felodipine, isradipine, and nitrendipine behaved as potent T-type channel blockers and were almost tenfold more potent than amlodipine and nifedipine [77]. The ability of selected DHPs to significantly inhibit T-type channel activity has relevant pathophysiological implications in the kidney. It has been shown, indeed, that whereas L-type Ca^2+^ channels are preferentially located at the afferent arterioles, T-type channels are highly represented also at the efferent arterioles where they control vascular tone [78–80]. Specifically, by immunocitochemistry Poulsen et al. [80] showed that CaV3.1 is the prevalent T-type isoform in human glomerular vasculature. Consistent with the location of T-type but not L-type Ca^2+^ channels in the efferent arterioles, “pure” L-type blockers only dilate afferent arterioles whereas T-type blockers like mibefradil dilate both afferent and efferent arterioles [81–82] (Fig. 1). Thus, filtration pressure, tends to increase when a pure L-type channel blockade is applied though this effect may be masked by the fall in systemic pressure [83–86]. Conversely, it is markedly reduced by T-type channel blockers [79]. Therefore, according to the hyperfiltration theory [87], which states that glumerular hypertension is a causative factor for the progression of chronic kidney disease (CKD), drugs with T-type channel blocker properties are expected to display nefroprotective effects [88] and could be helpful in delaying CKD progression [89]. Importantly, it has been observed that DHPs with a relevant T-type channel blocking activity exert favorable effects on the progression of CKD both in preclinical models of this disease and in humans. For instance, this has been reported for efonidipine [90–97], benidipine [98–104] and manidipine [105–111].

Non-hemodynamic effects may have a role in conferring nefroprotective properties to T-type blocking DHPs. T-type blockade [79]. Specifically, T-type channels control aldosterone synthesis in adrenocortical cells [112–113] and T-type blocking DHPs decrease aldosterone synthesis in vitro [114–115] (Fig. 1) and circulating aldosterone concentrations in hypertensive patients [91, 116–117]. In addition, T-type channels seem to control mesangial proliferation [118]. Intriguingly, it has been reported that efonidipine significantly decreases tubulo-interstitial fibrosis in an experimental model of chronic unilateral ureteral obstruction in the rat, a pharmacological effect that has been attribute either to the decrease of aldosterone concentrations caused by these drugs or to their antioxidant properties [119].

Considering the rich innervation by the noradrenergic system of both afferent and efferent arterioles, it is expected that they are both vasodilated upon orthosympathetic pharmacological blockade. As a matter of fact, DHPs with combined N- and L-type Ca^2+^ channel blocking properties showed similar effect in a number of preclinical models in vivo and in vitro [120–123] and some evidence that they could exert nefroprotective effects also in human patients have been reported [124–125] (Fig. 1). However, the entity of this effect seem to be considerably smaller than those of combined T- and L-type blocking DHPs as showns by the few comparative studies available. For instance, benidipine performed better in nefroprotection than cinildipine or amlodipine [126–127].

A mixed T- and L-type blockade could be a much more effective pharmacological approach than pure L-type blockade also in other clinical conditions. For instance, T-type channels are implicated in cardiac hypertrophy and in preclinical models mixed T- and L-type Ca^2+^ channel blockers proved to be effective as antiremodelling agents [128–131] (Fig. 1). Concluding this section we would like to emphasize thanks to a small loss of selectivity, DHPs acting also on T-type channels acquire relevant new pharmacological properties and, paradoxically, tissue-selective effects.

### C- FURTHER MECHANISMS FOR SELECTIVE EFFECTS ON SPECIFIC VASCULAR BEDS?

While the data reported in the previous section explain how some DHPs and not others may exert specific pharmacological effects in specific tissues, many pieces of information are still missing and we still do not have a definite explanation for other tissue-selective effects. However, a number of additional factors could be involved. In particular, it is now clear that the Ca_V_1.2 L-type Ca^2+^ channel family is much more complex that initially believed and that it encompasses much more members than just a cardiac and a smooth mucle cell isoform. As the Ca_V_1.2 gene is composed by 55 exons, 19 of which can be alternatively spliced, theoretically 219 Ca_V_1.2 L-type channel variants could be generated and approximately 40 of them have been actually identified [132]. The possibility that different splicing variants could be differently distributed in blood vessels of different tissues appears intriguing also considering that something similar has been demonstrated for other ion channels like voltage-gated K^+^ channels [133]. Unfortunately, a detailed mapping of their distribution of in different vascular beds is still missing and we ignore whether they differ in DHP sensitivity as well. However, we would like to mention that, as we mentioned above, Liao et al. [18] identified a splice variant that differs from Ca_V_1.2 because it lacks the 33 exon and showed that it activates at more negative potentials than Ca_V_1.2b, generates a window current and displays state-dependent block by nifedipine.

Another possibility that remains unexplored is that the Ca_V_1.3 channels subunit could be differentally expressed in different vascular compartments. It has been demonstrated, indeed, that this Ca^2+^ channels subtype, which, as reported above, differs from Ca_V_1.2 in its sensitivity to DHPs, is expressed in vascular smooth muscle cells but its distribution in different vascular beds has not been systematically explored yet [134].

Finally, T-type channels are also expressed in other vascular districts besides the kidney such as the brain resistance vessels [134–136] and the pulmonary arterioles [137–138]. The pharmacological and clinical implications of these observations are still unclear and will represent, indubitably, a rich area for future investigations.

## III. STRUCTURAL BASIS OF DHP FUNCTIONAL DIVERSITY

The evidence reported in the previous section suggests that the functional diversity of DHPs could be determined by subtle differences in the affinity either for channels other than Ca_V_1.2 or, possibly, for different isoforms of Ca_V_1.2 channels. They also raise, however, the question of how molecules with a similar structure could display such differences in target affinity. All the DHPs share, indeed, a similar basic structure consisting of an N-heterocyclyc pyridine ring bearing a phenyl substituent on its para-position. However, while the general three-dimensional structure of the molecule is believed to be preserved, both the N-heterocyclic ring and the benzene ring can be substituted in different positions giving rise to structurally different compounds (Fig. ). Since the early ‘80s it has been known that the nature of the substituents both on the heterocylic and on the benzene ring affect potency and tissue selectivity of DHPs. Specifically, using the Hansch analysis for Quantitative Structure-Activity Analysis (QSAR) Rodenkirken et al. [139] showed that the position of phenyl ring substitution affects the cardiodepressant potency of DHPs in the cat papillary muscle preparation. In particular, the DHPs whose phenyl ring is substituted in position ortho are the most cardiodepressant, those with substituents in position para the less cardiodepressant whereas metha-substituted compounds rank in-between [139]. The effect of the phenyl-substituents depends also on steric factors since the cardiodepressant activity increases with the width of the substituent. Similar results were obtained when the potency in relaxing guinea pig ileal preparations was measured [140].

While phenyl ring substitutions seem to affect mainly DHP potency, ester chain substitutions on the heterocylic ring influence not only the potency but also the selectivity of these drugs. In particular, vascular selectivity seems to increase with the size of the substituent. So niludipine [141] and nisoldipine [142], not only have much bigger side chain substituents on the heterocyclic ring but also a much higher vascular selectivity than nifedipine. Similarly, benidipine [143] and nicardipine [144], two DHPs with very large side chains, have a very favorable pharmacological profile with high vascular selectivity. Kojda et al. [145] explored the effect of lengthening the side chain ester substituents in nitrendipine derivatives and found that a small increase in side chain length (up to 3-I-propil-ester) determines an increase in vasodilator potency which did not correlate with the lipophylicity but with the charge of the side chain.

The substitution of the heterocyclic ring on position 3 and 5 has also another important structural consequence which can affect DHP potency and selectivity: whenever the residues on these two positions are not identical the DHP molecule bocomes chiral. In fact, the carbon 4 becomes a chiral center and two different optical enantiomers will exist for this DHP molecule. Starting from the observation that verapamil, a calcium channel blocker belonging to the family of phenylalkilamine, is also a chiral molecule and that its two enantiomers display a different potency in blocking L-type calcium channel [146], a few years after the synthesis of the first asymmetrically substituted DHPs, Towart et al. [147] looked at the potency of the enantiomers of a series of chiral DHPs. They found that exactly as in the case of verapamil, the two enantiomers of chiral DHPs display markedly different potencies. Since there, a number of studies confirmed and extended this observation. Clear differences in the potency of the two optical enantiomers of chiral DHPs have been described in a number of studies. For example, Shibanuma et al. [148] showed that two enantiomers of nicardipine display a different potency in lowering the blood pressure in the dog. Amlodipine [149] and nimodipine [150] enantiomers differ in their ability to induce the relaxation of high potassium-precontracted aorta strips in vitro. Similarly, manidipine [151] and benidipine (Muto et al., 1988) enantiomers display a different potency in lowering the blood pressure in rats. Inagaki et al. [152] examined in anestesized dogs the pharmacological activity of a the optical enantiomers of barnidipine, a DHP with two chiral centers, and found differences in the potency of its four stereoisomers with a potency order that agreed with that reported in vitro. A limited number of studies suggest that differences in potency between the different enantiomers of chiral DHPs similar to those observed in experimental animals could also exist in humans. For instance, Mikus et al. [153] examined the blood pressure lowering activity of the two nitrendipine enantiomers in healty volunteers and confirmed that the pharmacological activity of this drug is almost completely due to the S-enantiomer. Soons et al have obtained similar results [154] comparing the effects of the two enantiomers of felodipine and nitrendipine and of their racemic mixtures in young, healthy males.

Very limited data suggest that DHP chirality could also influence tissue selectivity. In particular, this has been observed for the two optical isomers of isradipine [155] because in anesthesized cats the S-enantiomer of this DHP is much more effective in lowering systemic pressure and heart rate than R-enantiomer, which, instead, is more potent in increasing subendocardial flux. Intriguingly, evidence of a role of the enantiomeric configuration in influencing tissue selectivity has been reported also for non-DHP chiral calcium channel blockers like verapamil [156].

Several mechanisms could determine the differences in potency and/or tissue selectivity observed among different enatiomers of the same DHPs. First, differences in potency in couples of DHP enantiomers could be determined by differences in the affinity for L-type channels. For instance, it has been shown that in radioligand studies the less potent enantiomer of nicardipine [157], manidipine [151], niguldipine [158] and benidipine [159] show a reduced affinity for these channels. As we mentioned above, some DHPs may also interact with Ca_V_ channel isoform different from Ca_V_1.2 L-type channels and this could be relevant in determining tissue-specific effects of these compounds. In this perspective, it interesting to emphasize that T-type channel blockade both by efonidipine and by benidipine is stereoselective with R-efonidipine and S-benidipine being much more potent than their enantiomeric counterparts [160–161]. This suggests that differences in tissue selectivity among DHP enantiomers could mirror differences in the choreography of the ion channels blocked by these drugs.

**Fig. 2:**
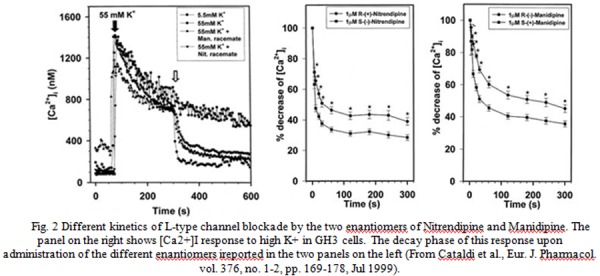
Different kinetics of L-type channel blockade by the two enantiomers of Nitrendipine and Manidipine. The panel on the right shows [Ca2+]I response to high K+ in GH3 cells. The decay phase of this response upon administration of the different enantiomers ireported in the two panels on the left (From Cataldi et al., Eur. J. Pharmacol. vol. 376, no. 1–2, pp. 169–178, Jul 1999).

It is also intriguing that relevant differences in the kinetics of Ca^2+^ channel blockade have been reported among different DHP enantiomers. For instance, great differences were observed in the time of onset and disappearance of the increase in coronary blood flow observed after the intracoronary administration of the four enantiomers of barnidipine [152]. While this represents only a very indirect indication of a different activity on L-type Ca^2+^ channels more direct evidences have bee reported. In particular, we used a microfluorimetric approach in fura-2 loaded pituitary GH_3_ cells to evaluate the kinetics of the inhibitory effect of the enantiomers of nitrendipine and manidipine on the increase in [Ca^2+^]_i_ elicited by membrane depolarization with an high K^+^ solution [162]. In control conditions this stimulus elicited a biphasic [Ca^2+^]_i_ response consisting of a first fast spike flowed by a long lasting plateau (Fig. 2). Nitrendipine and manidipine affected both these phases by lowering the spike and accelerating the decay of the plateau. However, in both cases, the more active S enantiomer almost exclusively blocked the fast spike without significantly affect the plateau phase channels whereas the less active R enantiomers only accelerated the decay of the plateau without affect the spike [162] (Fig. 2). In patch clamp experiments, this corresponded to a significantly longer time to maximal current inhibition in R as compared with S/enantiomers. Similar results were also obtained with the two optical isomers of lercanidipine [163] (Fig. 2). The differences that we observed could be possibly explained in terms of a differential affinity of the two optical isomers of the tested DHPs for open and inactivated Ca^2+^ channels according to the guarded receptor hypothesis similarly to what observed by Handrock et al. [164] for the two enantiomers of isradipine. By whole cell electrophysiology, they found, indeed, that the less potent enantiomer of isradipine, (-)-isradipine, preferentially blocks the Ba^2+^ currents flowing through L-type Ca^2+^ channels in the late phases of a depolarizing pulse when many channels are inactivated whereas the more active (+)-isradipine preferentially induces a block during the early phase of the pulse when the majority of the channels are in an open state.

## IV. CONCLUSIONS AND FUTURE PERSPECTIVES

In conclusion, though sharing a similar mechanism of action and a common main binding site on voltage gated Ca_V_1.2 Ca^2+^ channels, DHPs are actually an heterogeneous drug family with relevant differences in potency, tissue selectivity and pharmacokinetics. Here, we put the emphasis on the evidence that part of this heterogeneity originates from activities exerted on ion channels diverse from Ca_V_1.2. However, additional “unintended” pharmacological activities have been described in selected DHPs [165–166]. For instance, a number of marketed DHPs like nimodipine, felodipine and benidipine may have mineralcorticoid antagonistic properties [167–168]. Interestingly, this pharmacological activity is markedly dependent on the enantiomeric configuration [169] and it could be relevant in conferring to these drugs the ability to exert antiremodelling effects [170] (Fig. 1). Moreover, selected DHPs including amlodipine, azelnidipine, benidipine or cinildipine enhance the release of nitric oxide from the endothelium and exert antioxidant effects [171–179]. Additional pharmacological properties such as a combined α and β-adrenergic antagonistic activity [180–181] or NO-donor properties [182] may be conferred to DHPs by rationally designed modifications. In conclusion, modifications of the backbone of Ca_V_1.2 blocking DHPs can confer to these drugs additional, clinically relevant pharmacological activities. By improving our understanding of the mechanisms involved more and more effective new compounds can be designed so rejuvenating this evergreen drug family.

## Figures and Tables

**Table I:** CLASSIFICATION OF DHPs

	Drug	Half-life	Negative Inotropic Activity
1^st^ GENERATION	Nifedipine	Short	+
2^nd^ GENERATION	Felodipine, Nisoldipine	Long	+/−
3^rd^ GENERATION	Amlodipine, Lacidipine	Long	−
4^th^ GENERATION	Clevidipine	Very short	−
